# PHLPP2 suppresses the NF-κB pathway by inactivating IKKβ kinase

**DOI:** 10.18632/oncotarget.1774

**Published:** 2014-02-13

**Authors:** Nitin Kumar Agarwal, Xiaoping Zhu, Mihai Gagea, Charles L. White, Gilbert Cote, Maria-Magdalena Georgescu

**Affiliations:** ^1^ Department of Neuro-Oncology, MD Anderson Cancer Center, Houston, Texas; ^2^ Department of Endocrine Neoplasia, MD Anderson Cancer Center, Houston, Texas; ^3^ Department of Veterinary Medicine, MD Anderson Cancer Center, Houston, Texas; ^4^ Department of Pathology, UT Southwestern Medical Center, Dallas, Texas

**Keywords:** PHLPP, NF-κB, IKK, glioma, colorectal cancer

## Abstract

The NF-κB growth pathway is constitutively activated in many cancers but its activation mechanism is unclear in most cases. We show that PHLPP2 interacts with IKKβ kinase, decreases its phosphorylation and the subsequent NF-κB activation in cancer cells. PHLPP2 is progressively lost in glioma and colorectal cancer and acts as a bona fide tumor suppressor, depending on IKKβ expression in cells. Physiologically, IKKβ activation by growth factors requires the formation of the Bcl10-MALT1 ubiquitin-ligase complex leading to NEMO/IKKγ non-degradative ubiquitination and IKKβ phosphorylation. PHLPP2 opposes the formation of this complex through interaction with Bcl10 and competitive displacement of MALT1 from Bcl10. Conversely, PHLPP2 loss enhances Bcl10-MALT1 complex formation, NEMO ubiquitination and subsequent IKKβ phosphorylation, resulting in increased NF-κB-dependent transcription of multiple target genes. Our results reveal PHLPP2 as a new biomarker of cancer progression, and implicate it as major negative regulator of NF-κB signaling.

## INTRODUCTION

Studies of signaling pathways controlling cancer cell proliferation, migration and therapy resistance enabled better diagnosis and treatment by the discovery of progression markers and therapeutic targets. The transcription nuclear factor κB (NF-κB) pathway is one of the major pathways activated in cancer that promotes transformation, survival, proliferation, metastasis and chemoresistance of most types of cancer cells [1]. In unstimulated cells, NF-kB dimers are retained in the cytoplasm by the family of inhibitors of NF-κB (IκB). Pathway activation results after IκB phosphorylation by the IκB kinase (IKK) complex, leading to IκB ubiquitination and degradation, with subsequent nuclear translocation of NF-kB dimers. The IKK complex is the core element of the NF-κB cascade and consists of two kinases, IKKα and IKKβ, and a regulatory subunit, NEMO/IKKγ. Depending on the activating signal and cell type, two NF-kB pathways are distinguished: the canonical, activated by a wide variety of stimuli and depending on IKKβ and NEMO, and the noncanonical, activated only by a subset of stimuli and depending solely on IKKα. Although it has been shown that the NF-κB pathway is constitutively activated in various cancer types, the precise mechanism remains poorly understood.

The homologous pleckstrin-homology (PH)-domain leucine-rich-repeat protein phosphatases (PHLPP1-2) were recently described as survival/proliferation suppressors in various cancers [2-6]. Initially, they were reported as direct phosphatases for Akt [2, 3], but subsequent studies failed to detect their direct role in Akt dephosphorylation [5, 6]. Whereas PHLPP1 loss has been shown in a variety of cancers, PHLPP2 expression and role have remained largely unexplored [4-6].

We examined PHLPP2 in glioma and colorectal cancer (CRC), and documented its progressive loss. By undertaking a convergent systems approach to detect PHLPP2-associated proteins and PHLPP2-regulated proliferation/survival pathways, we identified PHLPP2 as a constitutive negative regulator of NF-κB signaling, through interaction and inhibition of IKKβ.

## RESULTS/DISCUSSION

### PHLPP2 is a tumor suppressor progressively lost in cancers

**Figure 1:**
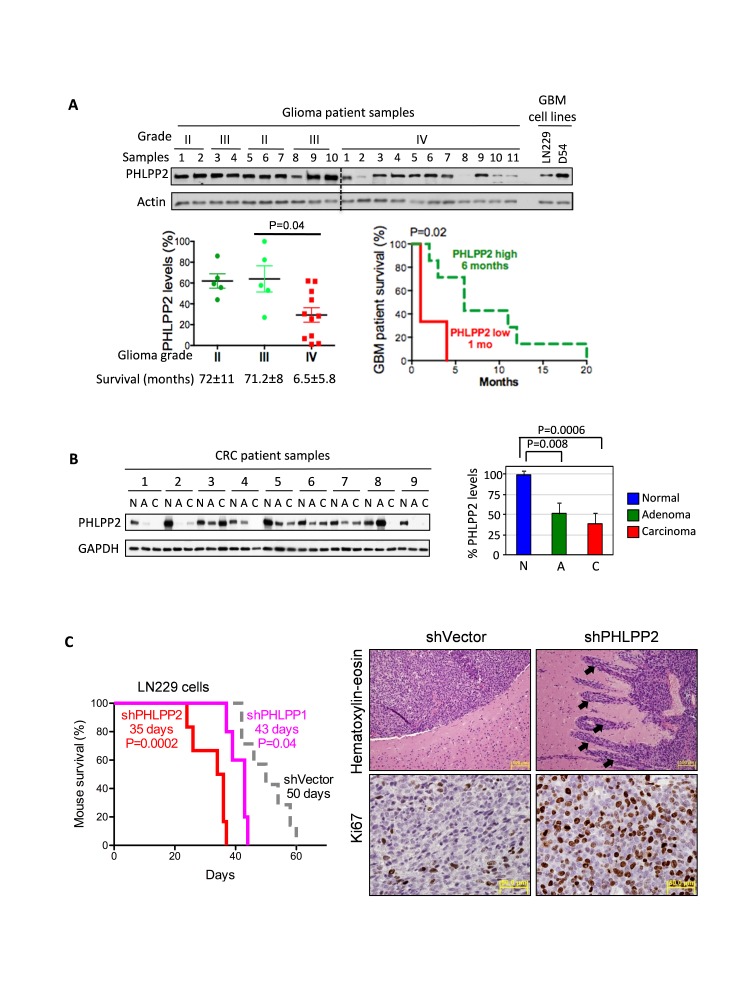
PHLPP2: progression marker in glioma and CRC (A) Immunoblot of protein extracts (8-10¼g) from grade II-IV glioma samples. Actin-normalized PHLPP2 levels and Kaplan-Meier survival analysis for glioblastoma patients with low (samples 2,8,10) and high (samples 1,3,4,5,6,7,9) PHLPP2 expression are shown. (B) Immunoblot of protein extracts (20¼g) from matched normal (N), adenoma (A), and carcinoma (C) CRC specimens. GAPDH-normalized PHLPP2 levels are shown as means±SEM. C. Kaplan-Meier survival analysis of 6-week-old SCID female mice (*n* = 5-7) inoculated intracranially with vector, PHLPP1- and PHLPP2-depleted LN229 cells. PHLPP2-depleted tumors show invasion into adjacent brain (arrows) and higher proliferation.

An initial analysis of PHLPP2 expression in tumors of increasing grade was performed on 21 glioma samples and showed decreased PHLPP2 expression in glioblastoma compared to lower grade gliomas (Fig.1A, left graph). PHLPP2 mRNA abundance showed also the same trend (Fig.S11). Survival analysis between glioblastoma cases with low versus high PHLPP2 expression showed significant shorter survival for PHLPP2 low-expressing cases (Fig.1A, right graph). Subsequent immunohistochemistry of a glioma TMA containing normal brain and glioma samples of increasing grade confirmed progressive PHLPP2 decrease (Fig.S1). To extend these findings to a different cancer type, we screened thirteen CRC resection specimens containing matched normal, adenoma and adenocarcinoma samples. A gradual decrease of PHLPP2 expression was observed in frozen specimens, with prominent PHLPP2 decline evident from early adenoma stages (Fig.1B). Similar results were obtained by immunohistochemistry (Fig.S2A), confirming previous data [4]. Gene expression profiles also revealed an important decrease of PHLPP2 transcripts in adenoma and carcinoma as compared to normal controls (Fig.S2B). Importantly, PHLPP1 mRNA levels showed minimal variation in these tumors. Collectively, these results reveal PHLPP2 as a cancer progression marker, with expression levels inversely correlated to tumor aggressiveness.

To establish PHLPP2 as bona fide tumor suppressor, we examined its effect on tumor growth and invasion. PHLPP2 expression was inversely correlated with cell proliferation in a panel of five glioblastoma lines (Fig.S3A). Stable PHLPP2 depletion in D54 and LN229 cells, led to increased cell proliferation (Fig.S3B). PHLPP2 knockdown in LN229 cells orthotopically inoculated in SCID mice induced highly infiltrative and proliferating tumors resembling human glioblastoma, and accelerated animal demise, more prominently than PHLPP1 knockdown (Fig.1C). Conversely, stable overexpression of Myc-tagged PHLPP2 in LN229 cells decreased proliferation and invasion, and delayed intracranial tumor growth and animal demise (Fig.S3C-E). These corroborated results clearly demonstrate PHLPP2 as a tumor growth and invasiveness suppressor.

### PHLPP2 and PHLPP1α interact with IKKβ and IKKα

**Figure 2:**
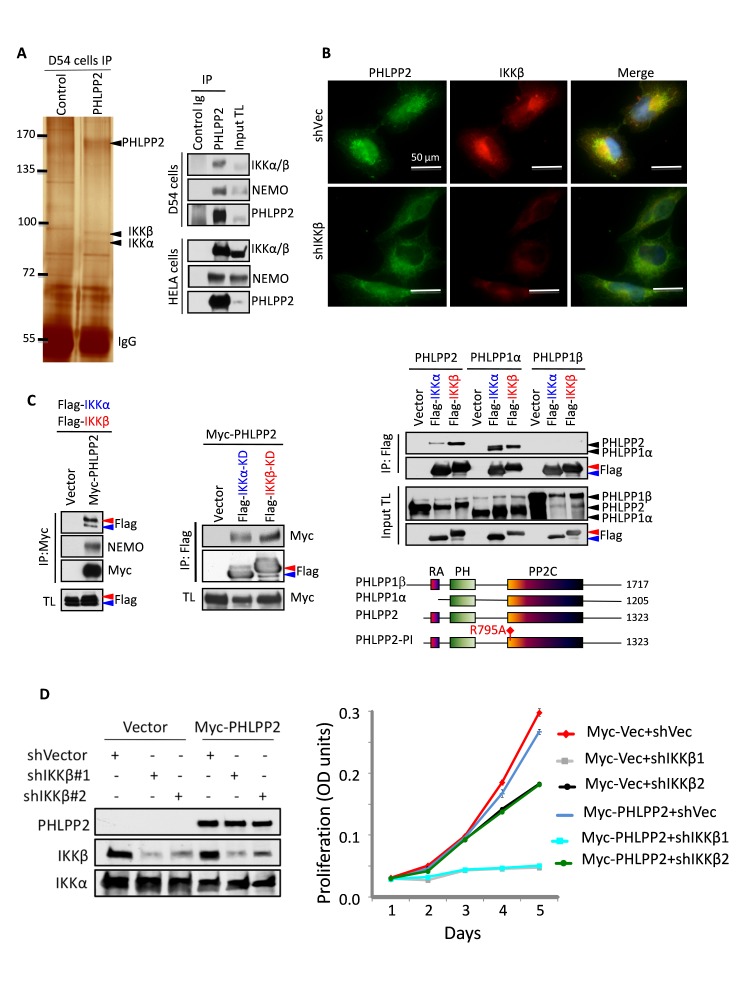
Association between PHLPP and IKK (A) Silver-stain and LC-MS/MS identification of IKKα/β immunoprecipitated (IP) with PHLPP2 (left), and confirmation of endogenous proteins associations (right). TL, total lysate. (B) Co-localization of endogenous proteins in LN229 cells expressing shRNAs vector (shVec) or IKKβ shRNA#1. (C) Co-immunoprecipitations of overexpressed proteins in 293T cells. The diagram indicates human PHLPP domains. RA, Ras-association; PP2C, protein phosphatase 2C-like. (D) IKKβ knockdown in LN229 cells abolishes the growth suppression effect of PHLPP2. Proliferation data are means±SD (n = 4).

To identify the signaling pathway controlled by PHLPP2, we explored putative interacting proteins by LC-MS/MS following immunoprecipitation of endogenous PHLPP2 from D54 cells (Fig.2A). In PHLPP2 immunoprecipitates, IKKα and IKKβ stood out with 41.1% and 37.4% sequence coverage, and 31 and 37 peptide hits, respectively. We verified this interaction by co-immunoprecipitating the endogenous IKKα/β/NEMO complex with PHLPP2 in two cell lines. Furthermore, PHLPP2 and IKKβ showed extensive perinuclear co-localization (Fig.2B).

We confirmed these findings with overexpressed Myc-PHLPP2 or untagged PHLPP1 splice isoforms and FLAG-tagged IKKα and IKKβ wild-type or kinase-dead (KD) forms in 293T cells (Fig.2C). Whereas both IKKα and IKKβ were detected in complex with Myc-PHLPP2, PHLPP2 showed stronger affinity for IKKβ. IKKα-KD and IKKβ-KD could also associate with PHLPP2, suggesting that their kinase function is dispensable for the interaction. Only PHLPP1α associated with IKKs, suggesting that the amino-terminal region of PHLPP1β inhibits this interaction. Unlike PHLPP2, PHLPP1α appeared to interact stronger with IKKα. The perinuclear co-localization of PHLPP2 and IKKβ was similarly confirmed with overexpressed proteins (Fig.S4). These data indicated that both PHLPP2 and PHLPP1α interact with IKK, with preferential formation of PHLPP2-IKKβ and PHLPP1α-IKKα complexes.

We examined the influence of PHLPP2-IKKβ in proliferation. IKKβ knockdown led to dose-dependent decreased proliferation (Fig.2D), effect previously observed in other cell types [7]. Nevertheless, the growth suppression induced by Myc-PHLPP2 in control cells was abolished in IKKβ-depleted cells, indicating PHLPP2 dependency on IKKβ for growth suppression.

### PHLPP2 inhibits IKKβ phosphorylation and represses NF-κB transcription

**Figure 3:**
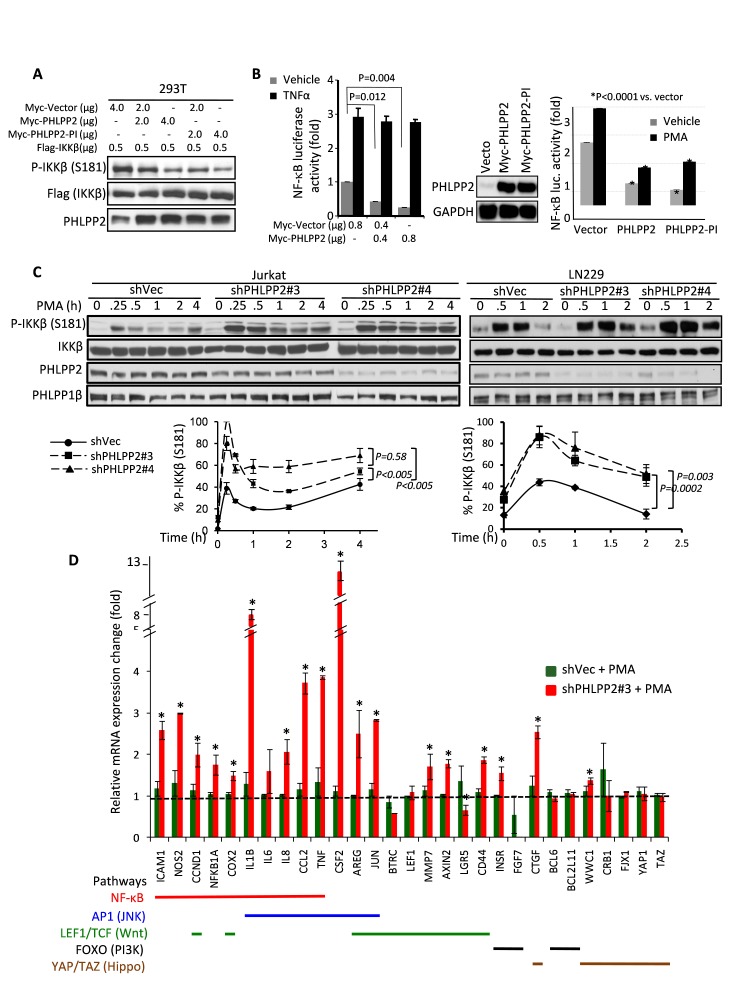
PHLPP2 suppresses IKKβ phosphorylation and NF-κB transcription (A) Co-expression of increasing amounts of PHLPP2 or PHLPP2-PI with IKKβ in 293T cells results in dose-dependent dephosphorylation of IKKβ. (B) NF-κB luciferase assay in 293T cells with Myc-PHLPP2 or Myc-PHLPP2-PI and TNFα or PMA treatment. Renilla-normalized NF-κB luciferase recordings are means±SD (n = 3). (C) Time-course PMA stimulation of indicated cell lines with PHLPP2 knockdown. Normalized IKKβS181/IKKβ levels are means±SD (n = 3). (D) Quantitative real-time-PCR microarray showing relative changes in transcript expression of indicated pathway target genes in PMA-treated control and PHLPP2-depleted LN229 cells. Gene expression was normalized to 60S-ribosomal-protein-L13a, and normalized values of unstimulated shVec transcripts were set to 1 (see Fig.S7).

The NF-κB canonical pathway is upregulated by various stimuli that lead to IKKβ activation by phosphorylation on Ser177 and Ser181 [1]. Co-expressing increasing amounts of PHLPP2 with IKKβ in 293T cells inhibited IKKβ-Ser181 phosphorylation (Fig.3A). We also showed a similar effect with PHLPP2-PI phosphatase-inactive mutant (see Fig.2C), ruling out a direct PHLPP2 phosphatase activity on IKKβ. Increasing amounts of PHLPP2 showed dose-dependent repression on NF-κB transcription in untreated cells but not in cells stimulated by TNFα (Fig.3B-left), indicating minimal interference with this activation mechanism. However, PHLPP2 and PHLPP2-PI efficiently suppressed NF-κB transcription following stimulation by phorbol 12-myristate 13-acetate (PMA), a surrogate of 1,2-diacylglycerol, which mimics NF-κB activation by growth factors (Fig.3B-right). These stimuli did not significantly impact the complex between PHLPP2 wild-type or mutant and IKK kinases (Fig.S5). These results indicated that PHLPP2 inhibits IKKβ phosphorylation and suppresses NF-kB transcription in a phosphatase-independent manner, particularly following PMA activation. Of note is that PHLPP2-PI is R-to-A mutant of the R795 phosphate-coordinating residue. In this configuration, PHLPP2 lacks three essential residues necessary for its catalytic activity, R795 that coordinates directly the phosphate, and two aspartate residues that coordinate the metal ion in the catalytic site, which are replaced by V800 and K985 in the PHLPP2 wild-type sequence [8]. Either one of these residues is essential for direct catalysis in the homologous PP2C phosphatase, as shown by mutagenesis studies [9, 10]. Moreover, analysis of wild-type PHLPP2 through the PHYRE threader program did not reveal that the mutated catalytic site residues are replaced by other substituting residues (courtesy of Dr. David Barford, Institute of Cancer Research, London, United Kingdom), predicting that even wild-type PHLPP2 does not have significant catalytic activity.

Alternatively, PHLPP2 knockdown by two independent shRNAs induced higher and more sustained IKKβ phosphorylation than control shRNA in two different cell lines (Fig.3C). Reconstitution of PHLPP2 expression in PHLPP2-depleted cells repressed IKKβ phosphorylation in both cell lines (Fig.S6), indicating that PHLPP2 specifically controls IKKβ phosphorylation.

The progressive PHLPP2 loss in tumors (Fig.1) prompted investigation of PHLPP2 knockdown consequences on a microarray of direct targets of transcription factors downstream of signaling pathways involved in tumor growth (Fig.3D and S7). Notably, the direct transcriptional targets of NF-κB and Jun kinase (JNK) pathways were significantly elevated by PHLPP2 depletion in PMA-stimulated cells (Fig.3D). Some of these targets, such as NOS2, cyclin D1, CSF2, AREG and Jun, showed also significant elevation in PHLPP2-depleted cells in the absence of PMA (Fig.S7). Other profiled pathways had lower activation patterns than NF-κB (Fig.3D). Overall, this comprehensive approach confirmed the proteomics a finding, highlighting that PHLPP2 growth effect is mediated through NF-κB, with possible contributions through JNK pathway.

### PHLPP2 prevents Bcl10-MALT1 complex formation and subsequent NEMO ubiquitination

**Figure 4:**
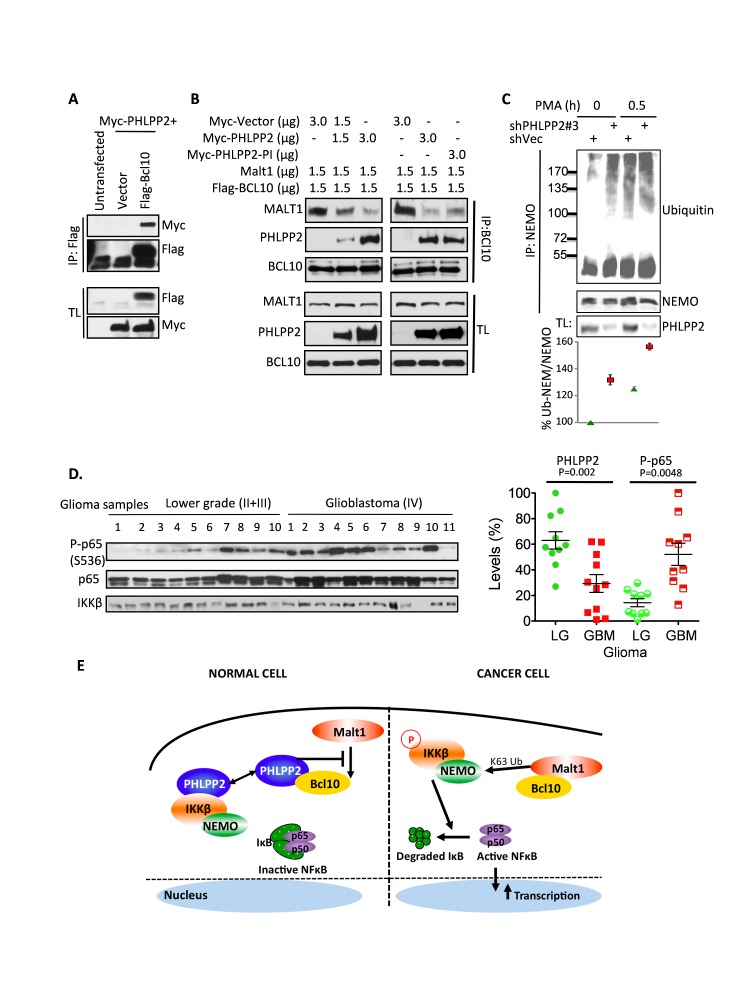
PHLPP2 interacts with Bcl10 to competitively displace MALT1 and prevent NEMO ubiquitintion (A-B) Co-immunoprecipitations in 293T cells of Myc-PHLPP2 with Flag-Bcl10 (A), and of PHLPP2 or PHLPP2-PI and MALT1 with Bcl10 (B). (C) Enhanced endogenous NEMO ubiquitination in PHLPP2-depleted LN229 cells. (D) NF-kB activation in gliomas (see Fig.1A) shown by quantifying Ser536-phosphorylated p65 subunit (P-p65) normalized to total p65 (P-p65/p65). (E) Model of NF-κB activation in cancer cells with PHLPP2 loss. P, phosphorylation.

IKKβ is phosphorylated by either TAK1 or MEKK3 kinases. NEMO, which directly associates with IKKβ, connects IKKβ to its kinase either by binding to nondegradative K63-linked polyubiquitin chains or by being itself polyubiquitinated with K63-linked chains. The complex inducing NEMO K63-linked-polyubiquitination is formed of Bcl10 and mucosa-associated lymphoid-tissue-1 (MALT1) protein, and is absolutely required for IKKβ activation following PMA stimulation [11-14]. Because PHLPP2 specifically inhibits NF-κB activation by PMA, we examined the interplay between IKKβ, PHLPP2 and Bcl10-MALT1. It was previously shown that IKKβ knockdown leads to dissociation of the Bcl10-MALT1 complex [13]. We confirmed this and found additionally that Bcl10 associates with endogenous PHLPP2, association increased by IKKβ knockdown (Fig.S8A) and not significantly altered by PMA treatment (Fig.S8B). PHLPP2 and Bcl10 co-localized perinuclearly, similarly to PHLPP2-IKKβ (Fig.S9). The PHLPP2-Bcl10 interaction was further demonstrated with overexpressed proteins (Fig.4A). Expressing increasing PHLPP2 or PHLPP2-PI led to dose-dependent dissociation of MALT1 from Bcl10 (Fig.4B). Moreover, PHLPP2 knockdown resulted in enhanced Bcl10-MALT1 complex formation (Fig.S10) and NEMO polyubiquitination that was further enhanced by PMA treatment (Fig.4C). These results indicated that PHLPP2 interacts with Bcl10 and inhibits the formation and ubiquitin-ligase activity of the Bcl10-MALT1 complex.

### Molecular-clinical correlations

The phosphorylation by IKKβ of the p65 NF-κB subunit on Ser536 is required for efficient transcriptional activity of NF-κB and is commonly used as marker for increased NF-κB activity in tumor samples [15]. The levels of phosphorylated p65 were significantly increased in glioblastoma as compared to lower grade tumors, and were inversely correlated with the PHLPP2 levels in these samples (P=0.014), supporting the mechanism of NF-κB activation in tumors by reduced PHLPP2 levels (Fig.4D).

With the advent of personalized cancer therapy, the discovery of markers for disease staging, progression and response to therapy is front line. We characterized PHLPP2 as a marker of tumor progression in two cancer types, glioma and CRC, and linked its tumor suppressor functions to the inhibition of the NF-κB pathway. In somatic cancers, the activation of NF-κB is common, but only some cases can be explained by known genetic alterations, such as *Bcl10* or *MALT1* rearrangements in MALT lymphomas, or mutations, polymorphisms and deletion of IκBα-encoding *NFΚBIA* gene in lymphoid malignancies but also in glioblastoma and CRC [16, 17]. In the majority of tumors with NF-κB activation, the mechanism of this activation is not known, and we propose that it involves PHLPP2 loss leading to increased IKKβ phosphorylation and activation (Fig.4E).

The control of IKKβ by PHLPP2 does not appear to involve direct dephosphorylation of IKKβ by PHLPP2, but rather relies on inhibition of NEMO ubiquitination by direct displacement of MALT1 from Bcl10 (Fig.4E). As NEMO ubiquitination is necessary for IKKβ activation, the net result of PHLPP2 inhibition looks similar to the inhibitory influences exerted by the deubiquitinase CYLD, which is the first deubiquitinase known to inhibit IKK activation [1]. The difference is that PHLPP2 appears to constitutively inhibit the formation of the ubiquitin-conjugating Bcl10-MALT1 complex through competitive displacement of MALT1. Further strengthening the inverse functional relationship between PHLPP2 and Bcl10-MALT1, the transcription profiling of glioma surgical samples of increasing grade showed significant PHLPP2 downregulation and opposite Bcl10 and MALT1 upregulation (FigS11).

The full significance of the constitutive interaction between PHLPP2 and IKKβ is not entirely clear. This interaction could function as a PHLPP2 reservoir, as forced IKKβ knockdown results in MALT1 displacement from Bcl10 most likely by PHLPP2 shift into the complex. However, it is possible that PHLPP2 may directly mask the phosphorylation sites on IKKβ or, alternatively, function as an anchor that brings other components into the larger IKK activation complex. PHLPP2 has been shown to interact with three deubiquitinases that have not been involved so far in NF-kB activation [18]. Further studies are necessary to elucidate whether PHLPP2 could recruit these deubiquitinases to limit the activation of IKKβ, similarly to CYLD.

In conclusion, the identification of PHLPP2 as a bona fide growth and invasion suppressor through inhibitory molecular interactions within the IKKβ and MALT1-Bcl10 complexes clarifies the contribution of PHLPP2 to tumor progression and provides a mechanism for the ubiquitous activation of the NF-κB pathway in tumors.

## METHODS

### Human Tissue Specimens

Glioma frozen samples comprise grade II oligodendroglioma, grade III anaplastic astrocytoma, oligoastrocytoma or oligodendroglioma, and grade IV glioblastoma. All patients were recorded in M.D. Anderson Cancer Center between 1991 and 2011. CRC frozen specimens with pathologist annotations as normal, deep tumor (carcinoma), and tumor-edge (adenoma), and paraffin-embedded specimens were obtained from the CRC Bank at M.D. Anderson Cancer Center. Surgeries were performed between 1992 and 2007, and no patient received prior therapy. These samples and the paraffin-embedded glioma tissue microarray (TMA) were previously described [5, 19, 20].

### Immunohistochemistry, immunoflorescence and histopathology

TMA immunohistochemistry with PHLPP2 antibody (Abcam, Cambridge, MA) and deconvolution immunoflorescence with PHLPP2 (Bethyl Laboratories, Montgomery, TX) and IKKβ (Santa Cruz Biotechnology, Santa Cruz, CA) antibodies, were performed as described [20]. The orthotopic tumorigenicity assay and tumor histopathology were described [21].

### Cells, plasmids, and functional assays

293T, HELA and glioblastoma cell lines LN229, LN18, D54, U251 and A172 were authenticated by the Cell Line Fingerprinting Core of Brain Tumor Center at M.D. Anderson Cancer Center using short tandem repeat profiling with GenomeLab Human Primer Set (Beckman Coulter, Brea, CA), as described [5, 22]. Jurkat T cells were provided by Dr. L. Nagarajan, M.D. Anderson Cancer Center. Plasmids and shRNAs are detailed in Supplemental Material. Transfections, retroviral infections, proliferation and matrigel invasion assays were described [22].

### Protein analysis and mass spectrometry

Cell lysis, immunoprecipitation immunoblotting and liquid chromatography-tandem mass spectrometry (LC-MS/MS) were described [20, 23]. Antibodies and ubiquitin immunoblotting are detailed in Supplemental material.

### Luciferase assay

The NF-κB-driven luciferase-reporter assay was described [24].

### Quantitative real-time PCR microarray

Based on commercially-available quantitative real-time PCR microarrays from SA Biosciences, Frederick, MD, we designed a growth pathway transcription target microarray [21] customized for us by SA Biosciences. Validated transcription targets and controls were arrayed in duplicate wells. Duplicate microarrays were run for each condition to ensure reproducibility. Briefly, 3x10^6^ control or PHLPP2-depleted LN229 cells were grown to 60-70% confluence, serum-starved for 16 h, and treated or not with 200ng/ml phorbol 12-myristate 13-acetate (PMA) (Thermo Fisher Scientific, Waltham, MA) for 5 h. Total RNA was isolated by using RNeasy Mini Kit (QIAGEN, Hilden, Germany), according to the manufacturer's instructions. 1¼g total RNA was reverse transcribed, using an RT^2^ First Strand Kit (SA Biosciences). The resulting cDNA was subjected to SYBR-Green based quantitative PCR (SA Biosciences) following manufacturer's protocol. RT2 Profiler PCR 96-well arrays were run on a Chrom4 system for real-time PCR detection (Biorad Laboratories). Data analysis was performed using the manufacturer's integrated web-based software package using cycle threshold (Ct)-based fold-change calculations.

### Statistical analysis

Kaplan-Meier survival analysis using Log-rank (Mantel-Cox) test was plotted with GraphPad Prism program (GraphPad Software, La Jolla, CA). ImageJ program was used for densitometric analysis (National Institutes of Health, Bethesda, MD). Data are representative of at least two or three independent experiments with similar results. Silencing data were derived from independent sets of lentiviral/retroviral infections with two different shRNAs. Numerical data were examined for normality of distribution and expressed as mean±SEM, unless mentioned otherwise. Two-tailed t-test with or without Welch's correction for variances significantly different and one-way analysis of variance were used to analyze the differences between groups. Statistical significance was considered for P<0.05. Confidence intervals for all tests were 95%.

## Supplementary Figures And Tables


