# Supplementary material for: PHLPP2 suppresses the NF-κB pathway by inactivating IKKβ kinase
Source: Oncotarget. 2014 Feb 13;5(3):815–23. doi: 10.18632/oncotarget.1774 (PMC3996652; doi:10.18632/oncotarget.1774)
Supplement: Supplementary file 1 [file oncotarget-05-815-s001.pdf]

SUPPLEMENTAL INFORMATION

SUPPLEMENTAL MATERIALS AND METHODS

Plasmids, retroviral infections and shRNA silencing. Human PHLPP1α and Myc-tagged PHLPP2 in pcDNA vector were described (9). Rat PHLPP1β (SCOP) was kindly provided by Dr. K. Shimizu, Tokyo University. FLAG-tagged IKKα and IKKβ wild-type and kinase-dead (K44A) constructs in pRK-5 mammalian expression vector were kindly provided by Dr. W. Liu (Tularik Inc., San Francisco, CA). Myc-tagged PHLPP2 and FLAG-tagged IKKb were further cloned in pCX_n (neomycin resistance) and pCX_b (blasticidin resistance) retroviral vectors, respectively. Human PHLPP2 shRNAs#3 (GCTAGGTATTTCCCAGGGAAA) and shRNA#4 (GCCTCGATACACTCTAC AAATT) were designed and cloned in the pSIREN-RetroQ retroviral vector (Clontech, Mountain View, CA). Lentiviral human IKKβ shRNAs IKKβ#sh1: TRCN0000018916 and IKKβ#sh2: TRCN0000018917 were from Sigma Mission shRNA (Sigma-Aldrich). MALT1 cDNA in pCDNA vector was kindly provided by Dr. X Lin, MD Anderson Cancer Center. Myc-tagged phosphatase-inactive PHLPP2, PHLPP2-PI, was engineered in pCDNA3 vector by R-to-A mutagenesis of the R795 phosphate-coordinating residue.

Protein analysis and antibodies. When indicated, cells were serum-starved for 16 h prior stimulation with 200ng/ml phorbol 12-myristate 13-acetate (PMA) (Thermo Fisher Scientific, Waltham, MA). To detect protein ubiquitination, cells were lysed in ice-cold lysis buffer containing 40 mM HEPES (pH 7.5), 120 mM NaCl, 1 mM EDTA, 10 mM sodium pyrophosphate, 10 mM sodium glycerophosphate, 50 mM NaF, 0.2% Triton X-100, and protease inhibitor cocktail (Roche, Basel, Switzerland) and lysates were normalized to equal protein concentration. SDS was added to lysates to a concentration of 1% and boiled for 5 min at 95°C. Supernatants were diluted tenfold with lysis buffer and incubated with antibody for 16 h at 4°C followed by incubation with 50% protein A/G ultra-link resin slurry (Thermo Fisher Scientific) for 2 h. Immobilized complexes were washed with ice-cold lysis buffer, eluted and subjected to immunoblotting.

The antibodies used were: IKKβ (T20), IKKα (B-8), NEMO/IKKg (FL-419 and 8330), IKKα/β (H470), GAPDH (0411), Bcl10 (333.1 and H197), Erk1 (C-16), Myc (9E10 and A14), Ubiquitin (P4D1), PTEN (A2B1) (Santa Cruz Biotechnology), EGFR (2232), P-IKKβ (Ser180/181) (2681), MALT1 (2494), P-Erk (9101) (Cell Signaling Technology, Danvers, MA), PHLPP1, PHLPP2 (Bethyl Laboratories), actin (Ac-15) (Sigma-Aldrich), Myc, N-Cadherin (Invitrogen, Carlsbad, CA) and FLAG (Rockland Immunochemicals Inc., Gilbertsville, PA).

SUPPLEMENTAL FIGURES

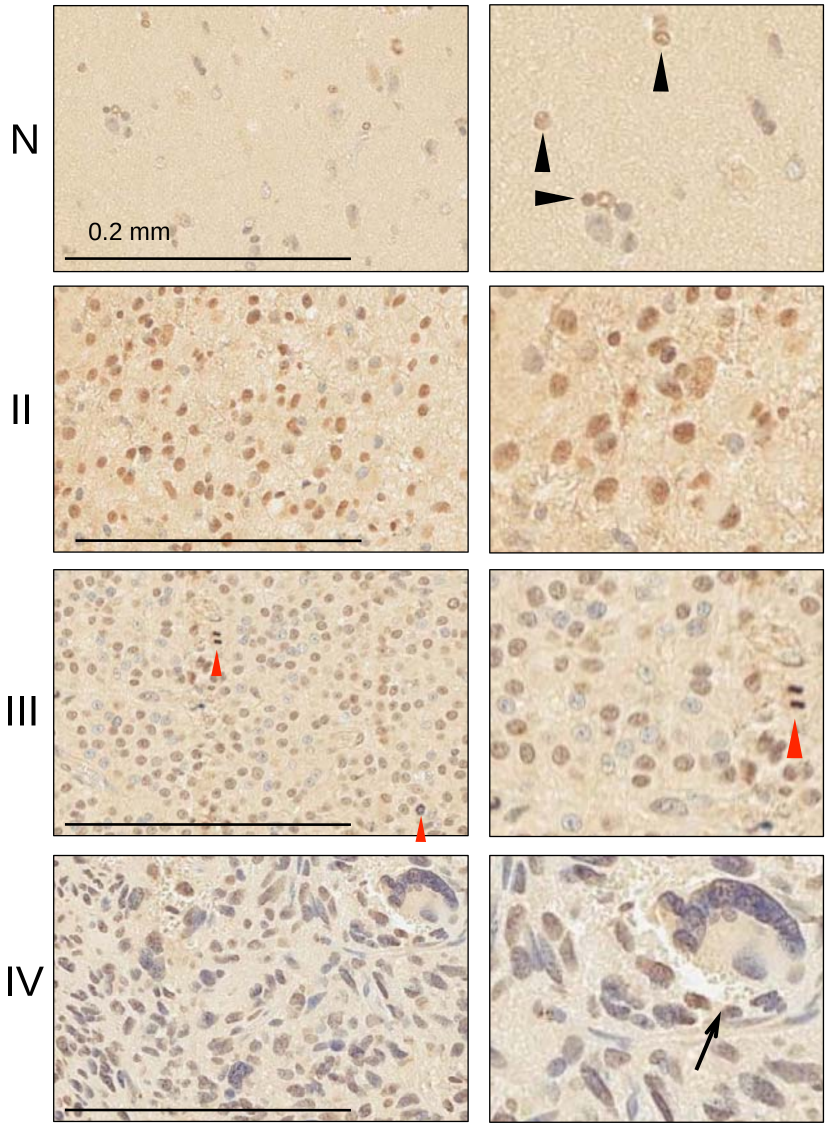

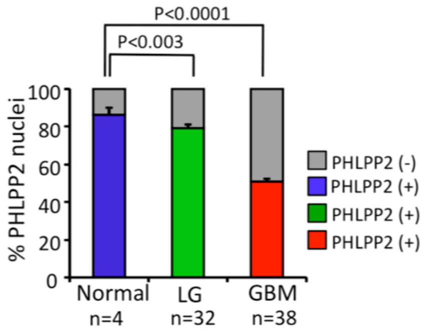

Fig. S1. Analysis of PHLPP2 expression in human glioma. PHLPP2 immunohistochemistry of a glioma TMA at lower (left) and higher magnification (right) shows nuclear and cytoplasmic staining of normal (N) and tumor cells. Normal oligodendroglial cells are indicated by black arrowheads. Red arrowheads indicate mitotic figures in an example of WHO grade III anaplastic oligoastrocytoma. A multinucleated giant cell is indicated by black arrow in a case of glioblastoma (GBM, WHO grade IV). The percentage, as means±SD, of PHLPP2-stained (+) or unstained (-) nuclei, was assessed in five random fields per specimen. The number of specimens analyzed is indicated. LG, lower grade glioma comprising grades II and III.

A

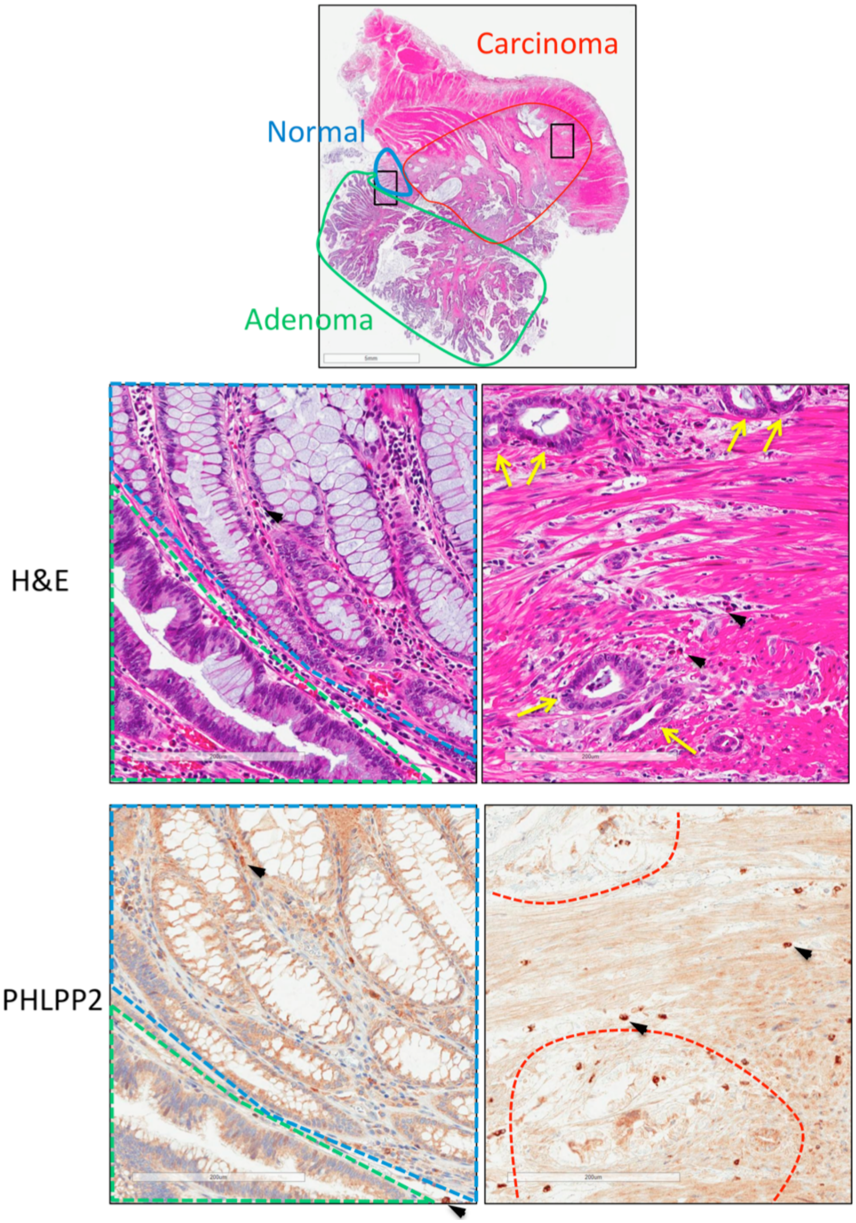

B

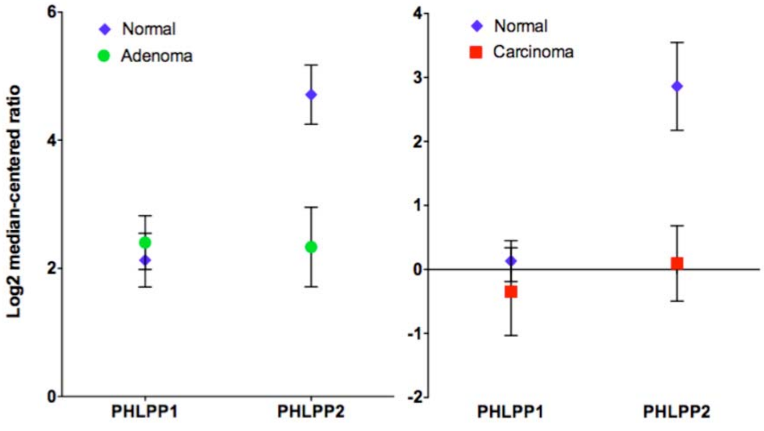

Fig. S2. Analysis of PHLPP2 protein and mRNA expression in CRC. **A.** Immunohistochemistry with PHLPP2 antibody of resection specimens (top) containing normal (blue contour), adenoma (green contour) and carcinoma (red contour) tissues. Boxed areas are shown below at higher magnification. Yellow arrows indicate adenocarcinoma gland structures invading the muscularis propria. Arrowheads mark eosinophils that strongly stain for PHLPP2. **B.** PHLPP1 and PHLPP2 gene expression in CRC datasets. The expression values publically available at Oncomine™ (www.oncomine.org) were derived from two datasets: the Sabates-Bellver Colon dataset (left graph), which contains 32 prospectively collected adenomas with matched normal mucosa from the same individuals, and a subset of 22 normal and 101 colon adenocarcinoma samples from the TCGA Colorectal dataset (cancergenome.nih.gov) (right graph). For the Sabates-Bellver Colon dataset, the select probesets that mapped to PHLPP1 (212719_at) and PHLPP2 (213407_at) were used for analysis (Affymetrix, Santa Clara, CA). For the TCGA Colorectal dataset, the probesets A_23_P89762 (PHLPP1) and A_23_P418235 (PHLPP2) were used. Plots were generated using the Oncomine-generated $\log_2$ median-centered ratio values obtained for each sample.

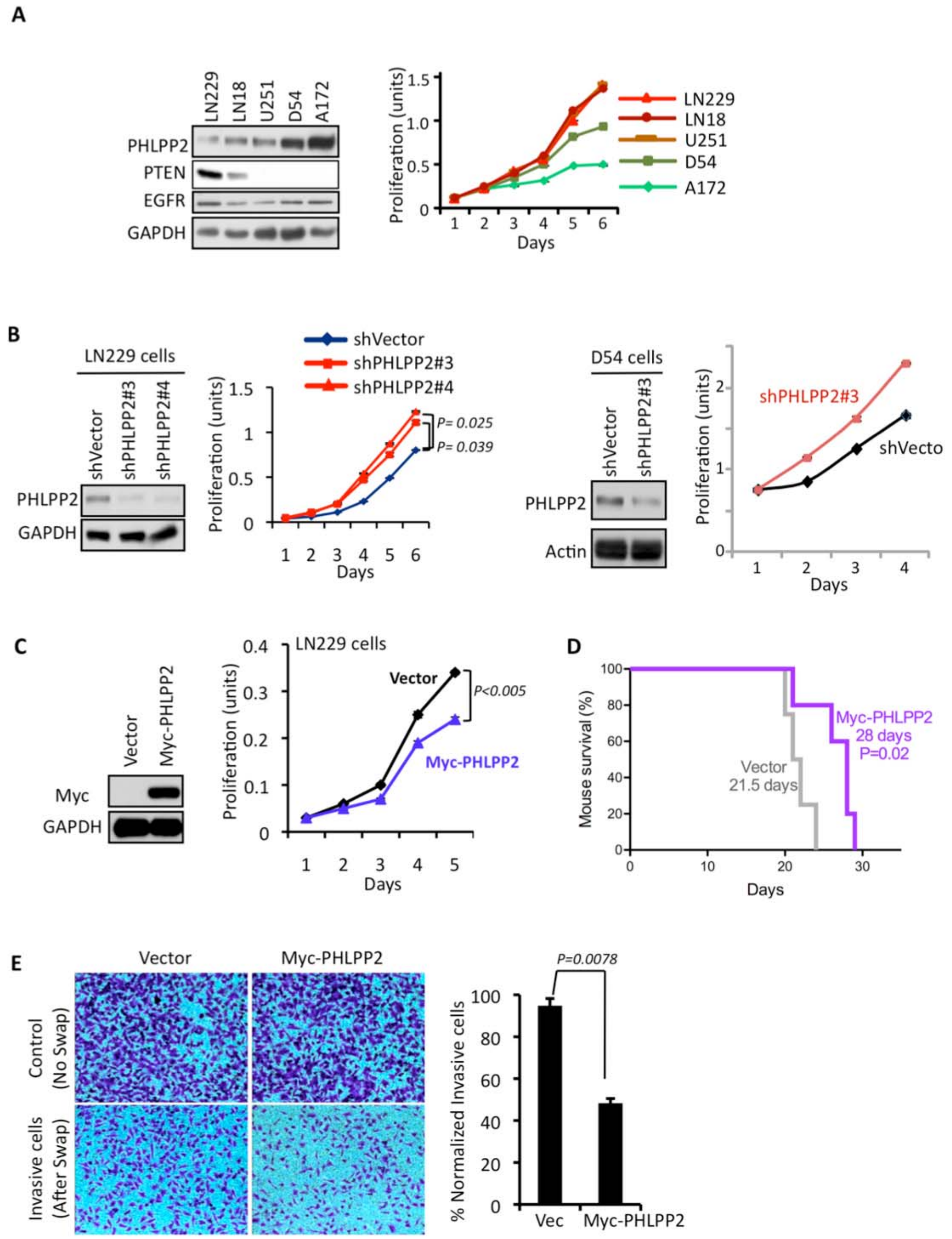

Fig. S3. PHLPP2 is a tumor growth and invasion suppressor *in vitro* and *in vivo*. **A.** Immunoblot of total cell extracts (30 μg) from the indicated glioblastoma cell lines shows PHLPP2 but not PTEN or EGFR levels inversely correlated with cell proliferation. Proliferation data are means±SD (n = 8). **B**. Proliferation of LN229 cells (left) expressing shRNA targeting vector (EGFP sequence) and two separate PHLPP2 hairpins. Protein lysates from these cells were analyzed by immunoblotting for PHLPP2 levels. Proliferation data are means±SD (n = 8). Proliferation of D54 cells (right) with PHLPP2 knockdown by shRNA#3 versus vector control. PHLPP2 levels were detected by immunoblotting. **C.** Immunoblot analysis and proliferation of LN229 cells expressing plasmids encoding vector or Myc-PHLPP2. **D.** Survival analysis of 6-week-old SCID male mice (n= 4-5) inoculated with the same cells as in (C). **E.** Matrigel invasion assay of the serum-starved LN229 cells from (C). The total number of cells plated on top of 8-μm pore size membrane inserts (upper panels) and the invasive cells that migrated on the bottom of the membrane inserts (lower panels) are shown. The invasive cells counted from five random fields were normalized to the total number of cells counted similarly, and represented as percentage of means±SD.

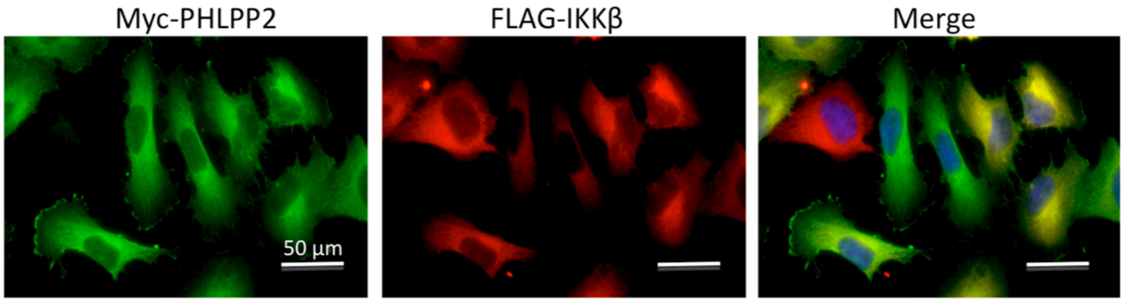

Figure S4. Co-localization of overexpressed PHLPP2 with IKKβ. Deconvolution immunofluorescence with PHLPP2 (green) and IKKβ (red) antibodies of LN229 cells co-expressing Myc-PHLPP2 and Flag-IKKβ. Note localization of overexpressed PHLPP2 preferentially to the cytoplasmic and membrane compartments and less to the nuclear compartment in LN229 cells, and strong co-localization with IKKβ in the cytoplasm in a perinuclear pattern, but not in the membrane.

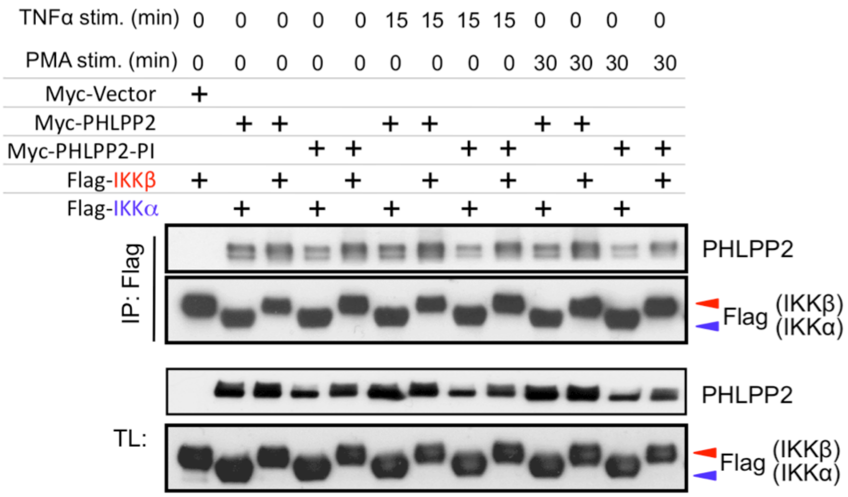

Figure S5. NF-κB stimulation by TNFα or PMA does not significantly alter the complex formation between PHLPP2 and IKKs. Co-immunoprecipitation (IP) of overexpressed Myc-tagged PHLPP2 wild-type or PI mutant with FLAG-tagged IKKβ. (red arrowhead) or IKKα (blue arrowhead) in 293T cells stimulated with TNFα (20 ng/ml) or PMA (200 ng/ml) for the indicated time periods.

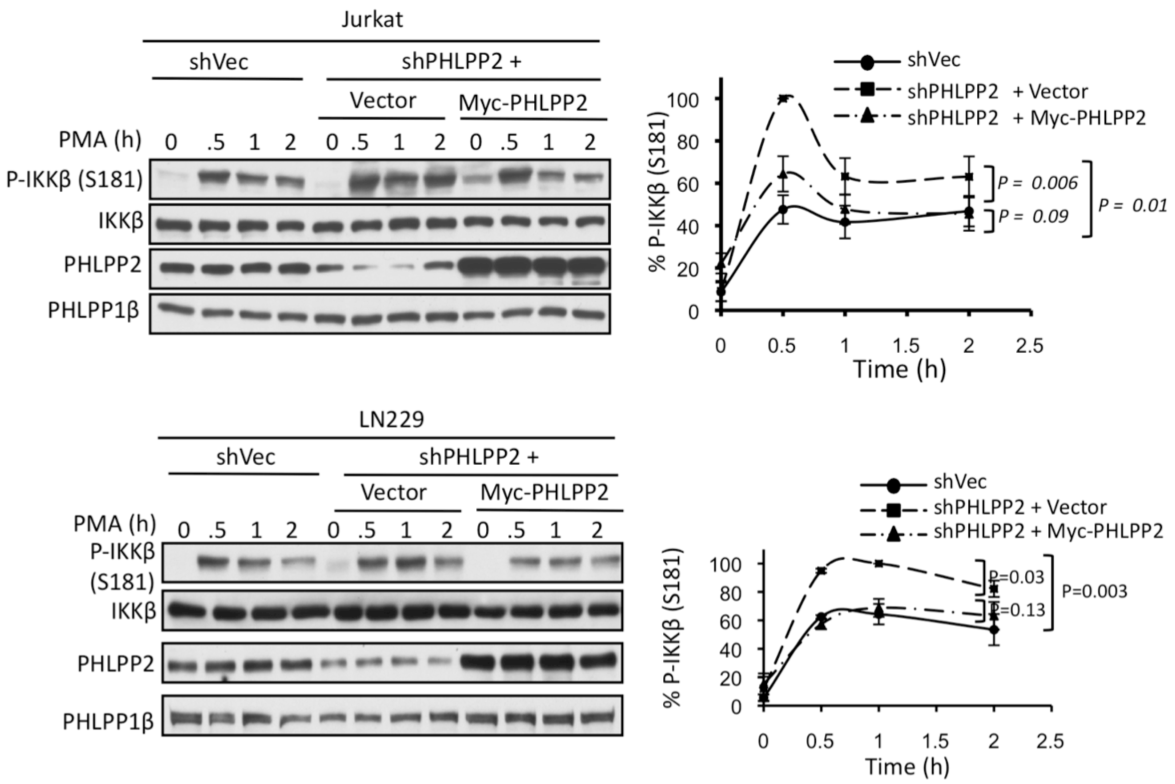

Figure S6. PHLPP2 specifically suppresses IKKβ phosphorylation. Time-course stimulation of serum-deprived Jurkat T cells and LN229 cells with PMA (200 ng/ml) for the indicated time periods shows higher IKKβ phosphorylation in PHLPP2-depleted cells compared to control (shVec) cells that is restored to control levels by stable expression of Myc-PHLPP2. Graphs show the normalized $IKK\beta^{S181}/IKK\beta$ levels as means±SD (n = 3).

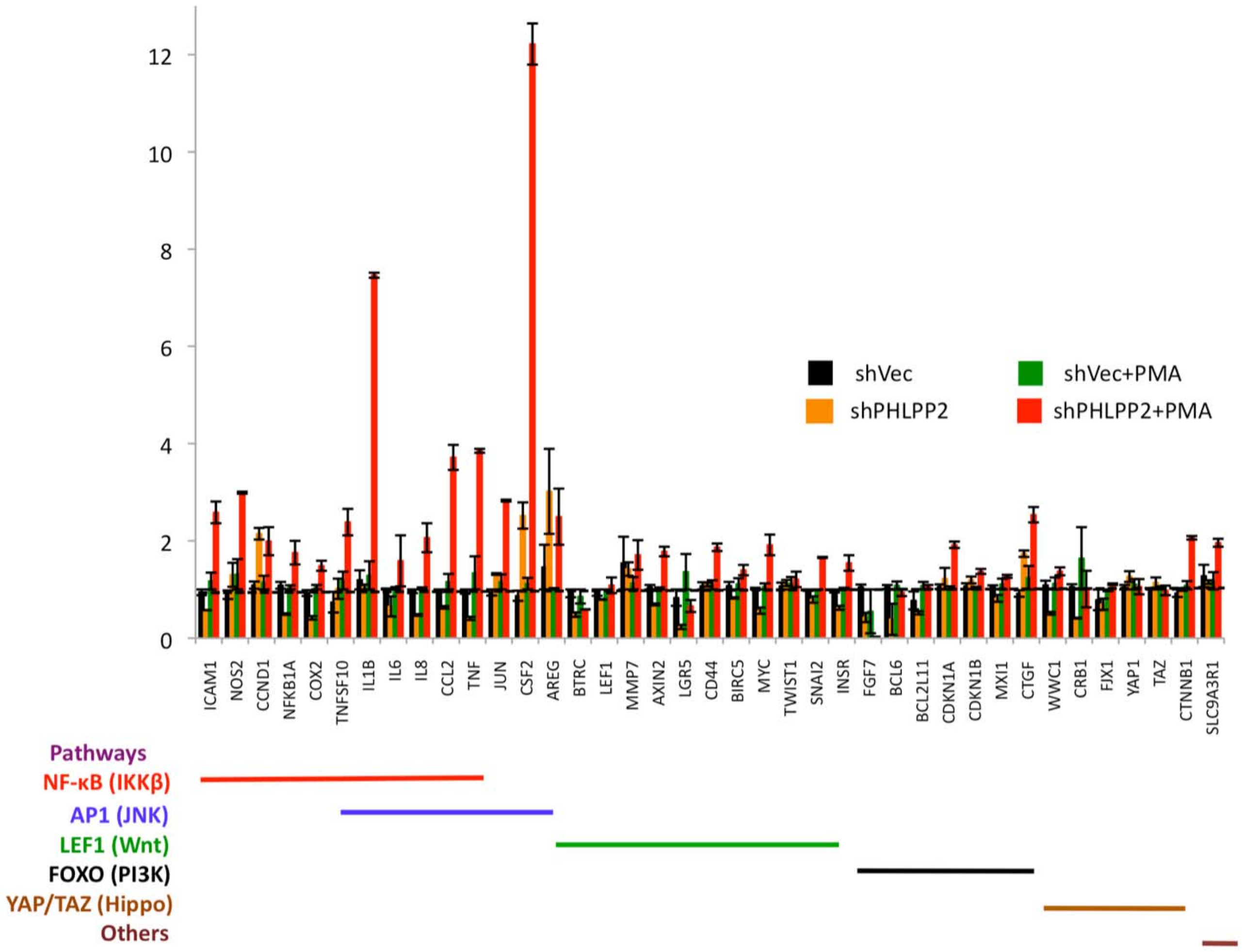

Fig. S7. PHLPP2 depletion synergizes with PMA stimulation to activate NF-κB target genes. Quantitative SYBER-green-based real-time PCR array showing relative changes in transcript expression of indicated signaling pathways target genes in untreated or PMA-treated control (shVec) and PHLPP2-depleted (shPHLPP2#3) LN229 cells. The expression levels of genes shown on the x-axis were normalized to the expression of the 60S ribosomal protein L13a.

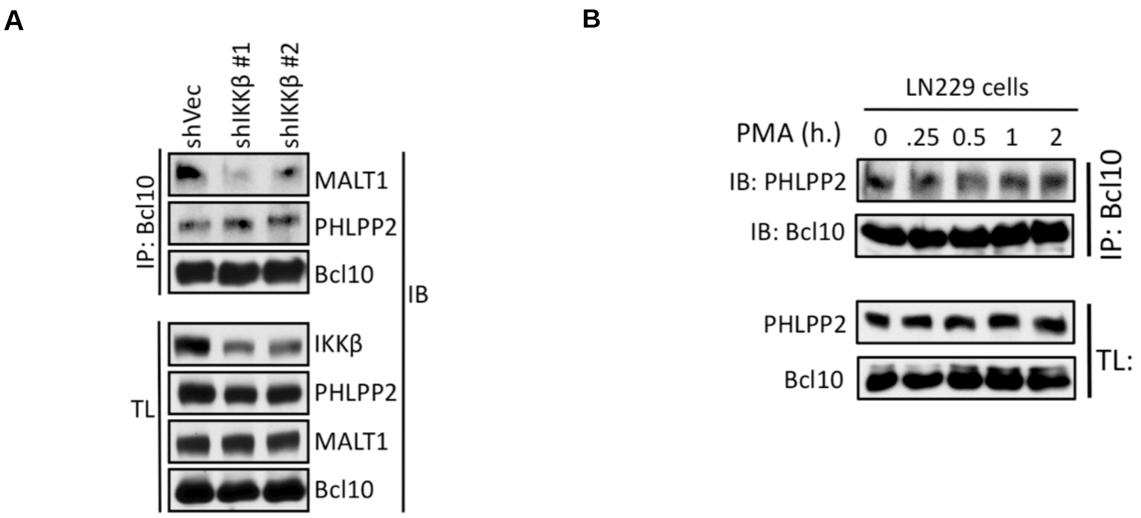

Fig. S8. PHLPP2 constitutively interacts with Bcl10 in vivo. **A.** Co-immunoprecipitation (IP) of endogenous PHLPP2 and MALT1 with Bcl10 in LN229 cells depleted of IKKβ by two shRNAs shows opposite occupancy of Bcl10 by MALT1 and PHLPP2. The diplacement of MALT1 from Bcl10 by IKKβ knockdown was initially observed by Wegener et al., Mol Cell, 2006. **B.** Co-immunoprecipitation (IP) of endogenous PHLPP2 with Bcl10 in LN229 cells treated with PMA (200 ng/ml) for the indicated time periods shows that the association between Bcl10 and PHLPP2 is unchanged by PMA treatment. TL, total cell lysates. IB, immunoblotting.

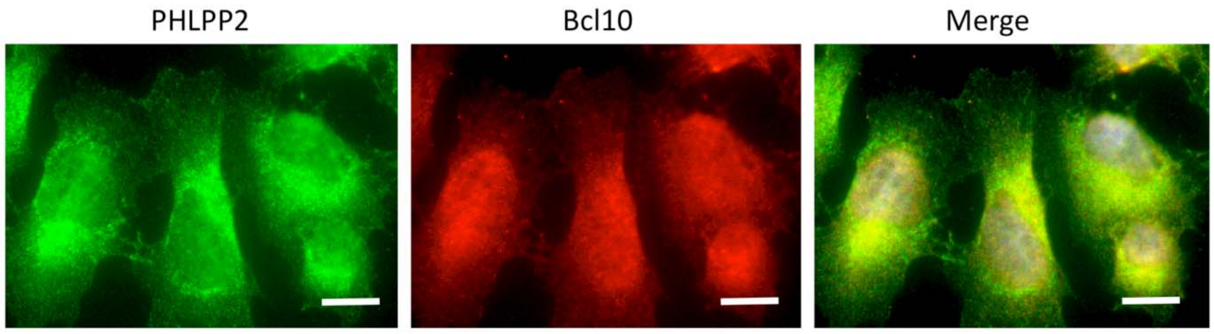

Fig. S9. Co-localization of endogenous PHLPP2 with Bcl10. Immunofluorescence analysis with PHLPP2 (green) and Bcl10 (red) antibodies in LN229 cells. Scale bar = 50 μm.

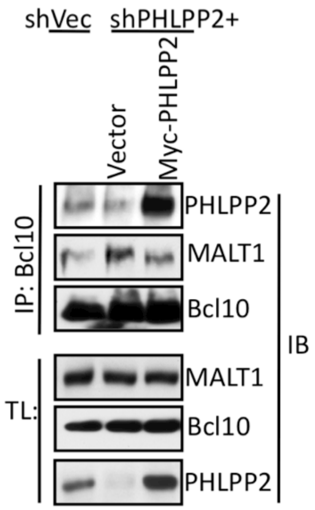

Fig. S10. PHLPP2 competes with MALT1 for Bcl10 occupancy. Co-immunopreciptation of PHLPP2 and MALT1 with Bcl10 in PHLPP2-depleted (shPHLPP2#3) LN229 cells with or without stable reconstitution of Myc-PHLPP2 by retroviral infection.

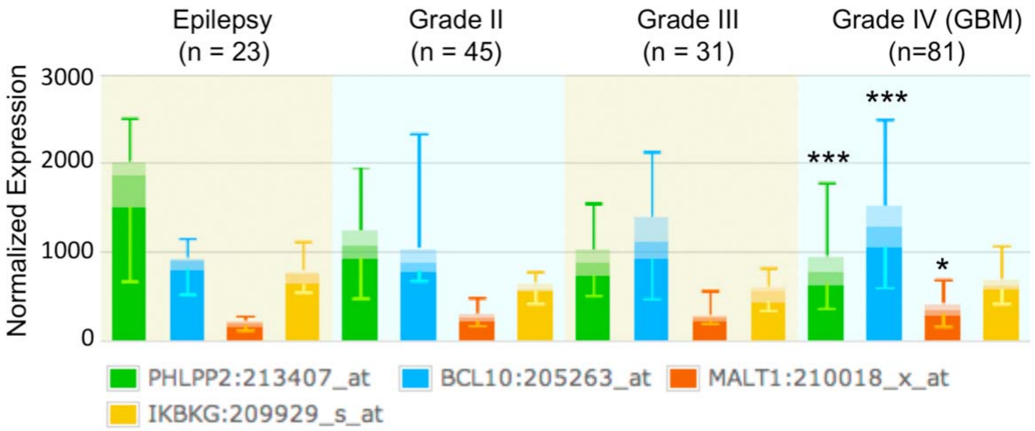

Fig. S11. Analysis of PHPPL2, Bcl10, MALT1 and NEMO (IKBKG) gene expression in glioma and epilepsy. Data derive from the Gene Expression Atlas analysis of dataset E-GEOD-4290 (referenced below). The E-GEOD-4290 dataset contains samples from 23 epilepsy brain samples, 45 grade II astrocytoma/ oligodendroglioma samples, 31 grade III anaplastic astrocytoma/oligodendroglioma samples, and 81 grade IV glioblastoma (GBM) samples. The bar graph shows the normalized expression values calculated from the raw Affymetrix HG-U133P2 array depicting the Maximum, Upper quartile, Median, Lower quartile and Minimum values for the 4 probes queried. Specific hybridization probes used for as a measure of each individual gene are indicated in the legend. Note significant downregulation of PHLPP2 and upregulation of Bcl10 and MALT1 in glioblastoma. P values of grade IV (GBM) vs.grade II: ***<0.0005; *<0.05. Note also opposite trend of these markers in epilepsy brain samples, shown for comparison.

Neuronal and glioma-derived stem cell factor induces angiogenesis within the brain. Lixin Sun, Ai-Min Hui, Qin Su, Alexander Vortmeyer, Yuri Kotliarov, Sandra Pastorino, Antonino Passaniti, Jayant Menon, Jennifer Walling, Rolando Bailey, Marc Rosenblum, Tom Mikkelsen, Howard A Fine. *Cancer Cell* :287-300 (2006)
